# Left Ventricular Myocardial Dysfunction Evaluation in Thalassemia Patients Using Echocardiographic Radiomic Features and Machine Learning Algorithms

**DOI:** 10.1007/s10278-023-00891-0

**Published:** 2023-09-21

**Authors:** Haniyeh Taleie, Ghasem Hajianfar, Maziar Sabouri, Mozhgan Parsaee, Golnaz Houshmand, Ahmad Bitarafan-Rajabi, Habib Zaidi, Isaac Shiri

**Affiliations:** 1 Department of Medical Physics, Iran University of Medical Sciences, Tehran, Iran; 2 Division of Nuclear Medicine and Molecular Imaging, Geneva University Hospital, CH‑1211 Geneva 4, Switzerland; 3 Rajaie Cardiovascular Medical and Research Center, Iran University of Medical Sciences, Tehran, Iran; 4 Echocardiography Research Center, Rajaie Cardiovascular Medical and Research Center, Iran University of Medical Sciences, Tehran, Iran; 5 Cardiovascular Interventional Research Center, Rajaie Cardiovascular Medical and Research Center, Iran University of Medical Sciences, Tehran, Iran; 6 Geneva University Neurocenter, University of Geneva, Geneva, Switzerland; 7 Department of Nuclear Medicine and Molecular Imaging, University of Groningen, University Medical Center Groningen, Groningen, Netherlands; 8 Department of Nuclear Medicine, University of Southern Denmark, Odense, Denmark; 9 Department of Cardiology, Inselspital, Bern University Hospital, University of Bern, Bern, Switzerland

**Keywords:** Thalassemia, Echocardiography, Cardiac magnetic resonance imaging, Machine learning, Radiomics

## Abstract

Heart failure caused by iron deposits in the myocardium is the primary cause of mortality in beta-thalassemia major patients. Cardiac magnetic resonance imaging (CMRI) T2* is the primary screening technique used to detect myocardial iron overload, but inherently bears some limitations. In this study, we aimed to differentiate beta-thalassemia major patients with myocardial iron overload from those without myocardial iron overload (detected by T2*CMRI) based on radiomic features extracted from echocardiography images and machine learning (ML) in patients with normal left ventricular ejection fraction (LVEF > 55%) in echocardiography. Out of 91 cases, 44 patients with thalassemia major with normal LVEF (> 55%) and T2* ≤ 20 ms and 47 people with LVEF > 55% and T2* > 20 ms as the control group were included in the study. Radiomic features were extracted for each end-systolic (ES) and end-diastolic (ED) image. Then, three feature selection (FS) methods and six different classifiers were used. The models were evaluated using various metrics, including the area under the ROC curve (AUC), accuracy (ACC), sensitivity (SEN), and specificity (SPE). Maximum relevance-minimum redundancy-eXtreme gradient boosting (MRMR-XGB) (AUC = 0.73, ACC = 0.73, SPE = 0.73, SEN = 0.73), ANOVA-MLP (AUC = 0.69, ACC = 0.69, SPE = 0.56, SEN = 0.83), and recursive feature elimination-K-nearest neighbors (RFE-KNN) (AUC = 0.65, ACC = 0.65, SPE = 0.64, SEN = 0.65) were the best models in ED, ES, and ED&ES datasets. Using radiomic features extracted from echocardiographic images and ML, it is feasible to predict cardiac problems caused by iron overload.

**Supplementary Information:**

The online version contains supplementary material available at 10.1007/s10278-023-00891-0.

## Introduction

One of the common forms of inherited anemia caused by a malfunction with hemoglobin synthesis is thalassemia [1]. Approximately 1.5% of people worldwide, according to a World Health Organization (WHO) estimate, may be thalassemia carriers [1]. Defects in the synthesis of hemoglobin chains occur in one of the forms alpha-thalassemia (reduction or lack of synthesis of alpha globin chains) and beta-thalassemia (decrease or absence of synthesis of beta-globin chains) [2]. Beta-thalassemia is a genetic disorder that leads to the incomplete synthesis of beta-globin chains and, eventually, hemolytic anemia [3]. Beta-thalassemia is a common genetic disorder worldwide which roughly 9% of thalassemia patients suffer from it [4]. Beta-thalassemia is seen in one of the forms encompassing beta-thalassemia major, beta-thalassemia intermedia, and beta-thalassemia minor [5].

Patients with thalassemia major require frequent blood transfusions due to severe anemia, and regular blood transfusions cause iron overload in these patients [5]. Iron overload can lead to heart problems (cardiomyopathy), liver and endocrine gland involvement [5, 6], osteoporosis, splenomegaly, chronic hepatitis, and delayed growth and sexual maturity in children [5]. Among the stated complications, heart failure caused by iron deposition in the myocardium is the leading cause of mortality in 71% of beta-thalassemia major patients [7]. Cardiomyopathy evoked by cardiac siderosis (iron overload) is the most common and life-threatening issue in B-thalassemia major patients [5, 8]. Moreover, the life span of these patients is limited [9]. Hence, early recognition of myocardial dysfunction can lead to initiating the iron chelation treatment in time [4] to reverse cardiomyopathy caused by iron deposition [10]. However, the initial diagnosis of heart failure patients is difficult, as the left ventricular function is preserved until the later stages of the disease in these patients [9].

Identifying and measuring iron deposits, especially in the heart, in patients with thalassemia major with frequent injections and patients receiving chelation treatments should be considered. There are several methods to evaluate and control iron overload. Serum ferritin level, as the broadest tool with the lowest cost in assessing the body’s iron concentration [11], may change under the influence of several conditions, including inflammation, infection, liver damage, and chelation treatments. Therefore, it cannot accurately measure iron overload [12, 13]. Another approach to testing iron overload is liver biopsy, an invasive procedure with risks and complications [10] that cannot analyze and identify cardiac iron deposits [14]. In addition, due to the non-uniform distribution of iron in the liver, the results obtained from the biopsy may not have the necessary accuracy [12, 15], such that in a situation where a significant liver iron overload is seen, cardiac iron overload may not be evident [15]. On the other hand, a myocardial biopsy lacks the sensitivity required to identify cardiac iron deposits due to [14] the fragmentation of iron deposits [16].

Cardiac magnetic resonance imaging (CMRI) is a non-invasive method to evaluate cardiac iron deposits. Moreover, T2*CMRI is an outstanding and non-invasive technique in evaluating myocardial iron deposits [17–20]. T2* relaxation time is a parameter in MRI that shows the speed of signal decay in tissues with an iron overload [21]. As cardiac iron deposition increases, T2* value in MRI decreases [4, 6, 8, 14, 21, 22] because iron disrupts the magnetic field’s uniformity and speeds up signal decay [14, 16, 23]. Despite the advantages of this technique, CMRI is costly and is not generally available in all medical centers [8–10, 18]. In addition, some patients are claustrophobic or cannot undertake MRI due to having metal implants or devices such as pacemaker [18].

Echocardiography is a method to identify cardiac dysfunction caused by iron overload in patients with thalassemia major. In studies conducted by Abtahi et al. [4] and Khaled et al.[13], no significant relationship was observed between LVEF from echocardiography and cardiac T2*. One of the most used imaging procedures in cardiology is echocardiography; however, the correct interpretation of its findings relies on the user’s experience and knowledge [24]. Artificial intelligence, machine learning (ML), and radiomic analysis could be potentially efficient in this era. Radiomics involves the extraction with high throughput of quantitative features from digital images to construct predictive or diagnostic models [25–28]. Radiomics plays an important role in medical image analysis, especially in cancer and cardiac imaging [29–34]. Radiomics extracts quantitative characteristics from medical images and probed to hold significant promise in predicting factors, such as lesion malignancy, prognosis, and treatment response prediction (e.g., tumor response and recurrence risks) [35]. Radiomic biomarkers can be associated with clinical observations [35] and can be utilized to estimate survival rates [36].

In a study conducted by Baessler et al. [37], the ability of texture analysis of CMRI images to distinguish between myocardial problems and a healthy heart was investigated in non-contrast cine images. Also, Cetin et al. [38] demonstrated the ability of radiomics of CMRI images to detect myocardial structural changes in cases with hypertension but apparently healthy. Additionally, the use of artificial intelligence in cardiovascular imaging is expanding quickly [24]. Artificial intelligence has the ability to reduce human errors and facilitate the identification and prediction of diseases and the decision-making process [24]. Numerous studies highlighted the effectiveness of radiomics in identifying changes in myocardial tissue in patients with hypertrophic cardiomyopathy and detecting cardiovascular changes in hypertensive patients with healthy hearts using CMRI [38, 39]. However, echocardiography has certain advantages over CMRI, including its availability, outpatient nature, non-invasiveness, and portability [24]. Therefore, it is crucial to empower echocardiography in identifying cardiovascular problems using radiomic features, as it is capable of offering the abovementioned benefits.

The main objective of this study is to utilize echocardiography radiomic features and ML algorithms to identify individuals at risk of developing cardiac issues in the near future due to excessive iron accumulation in their hearts. These individuals have been categorized based on CMRI T2* results.

## Materials and Methods

### Study Design and Dataset

**Fig. 1:**
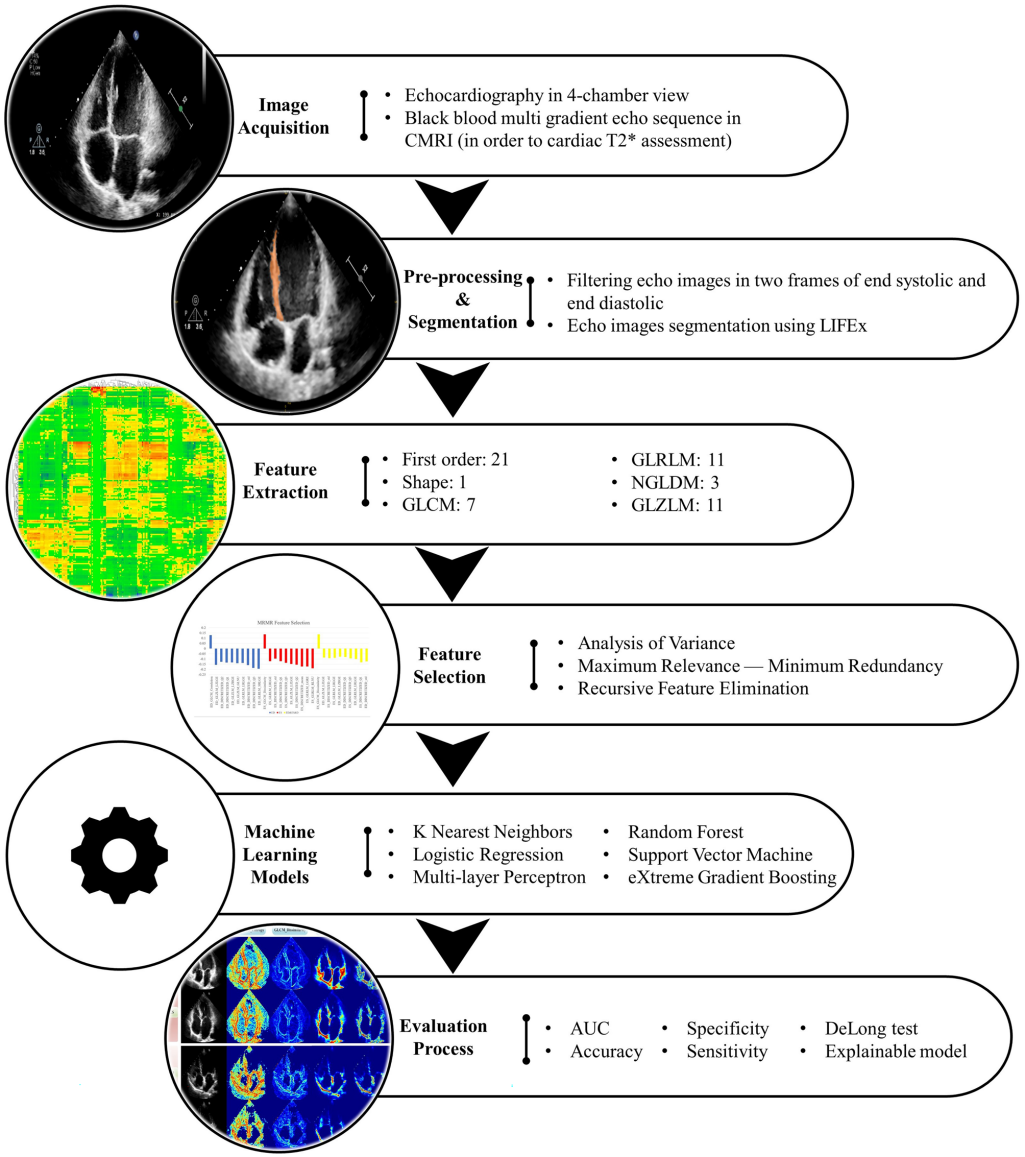
The framework of the current study

The framework of the study is shown in Fig. 1. In the following, each part of the workflow will be elaborated.

Ninety-one patients with an age range of 15 to 50 years (31 ± 7.56 years) were enrolled in this study. Forty-four patients with thalassemia major (age: 30.40 ± 7.05 years) whose heart condition was examined and followed up at a maximum interval of 6 months by echocardiography and CMRI were included in the study. Patients had normal echoes (LVEF > 55%) and T2* ≤ 20 ms in CMRI. In addition, 47 people (age: 33.23 ± 7.75 years) and conditions as thalassemia patients who had an echo and CMRI tests conducted at a maximum interval of 6 months and those with LVEF > 55% and T2* > 20 ms were entered into the study as the control group. Table 1 shows the characteristics of the population investigated, and in Fig. 1S, the CMRI image of two cases, including one T2* > 20 and one T2* ≤ 20, is presented. Patients with valvular heart problems, congenital heart diseases, infectious diseases, hypertension, liver failure, diabetes, and kidney diseases, as well as patients who used drugs that changed myocardial function, were excluded from the study.

**Table 1:** Demographic data of the studied patient population

Group	Number	T2* (mean ± SD)	Range of T2*	Age (mean ± SD)	Gender	Number	Age (mean ± SD)
Thalassemia patients (T2* ≤ 20 ms)	44	13.37 ± 6.25	3–20	30.40 ± 7.05	Male	20	29.70 ± 7.05
					Female	24	31.00 ± 7.03
Control group (T2* > 20 ms)	47	29.42 ± 6.33	21–45	33.23 ± 7.75	Male	19	32.05 ± 6.91
					Female	28	34.03 ± 7.75
*P* value		< 0.0001^a^		0.07^a^	0.78^b^		

*SD* standard deviation

^a^Student’s *t*-test

^b^Chi-squared test

### Image Acquisition

Two-dimensional M-mode and Doppler echocardiography (pulsed-wave Doppler, continuous-wave Doppler, colored Doppler) and tissue Doppler echocardiography were performed using the echocardiography system by the transthoracic method in supine. Left lateral decubitus positions for all subjects were performed to obtain a 4-chamber view. Within less than 6 months, patients with thalassemia major and those selected as the control group were subjected to CMRI examinations. MRI examinations were performed using a 1.5 Tesla scanner (Avanto-Siemens). To evaluate cardiac T2*, a black-blood multi-gradient echo sequence was obtained in short axis view with 8 echo times (TE) and 10 mm slice thickness. In addition, fast spin echo sequences were routinely performed to examine the morphology of the heart. Myocardial function was evaluated using cine CMRI protocols such as steady-state free precession (SSFP) with a slice thickness of 8 mm and a gap of 2 mm in the short and long axis (2 chambers, 3 chambers, and 4 chambers).

### Preprocessing and Segmentation

After obtaining the echo images in the video, the frames of the end-systolic (ES) and end-diastolic (ED), according to the patient’s electrocardiogram, were extracted from all the frames related to the 4-chamber view. Furthermore, filtration was also done on them due to the low quality and high Speckle noise. The linear statistical filter DsFlsmv, which had the best performance and had the most negligible impact on the radiomic analysis [40], was applied to the images to remove the noise. Finally, the filtered images (two frames of ES and ED) were used for segmentation. The ventricular septum in filtered echo images was segmented manually by an experienced echocardiographer and edited/verified by a specialist using LIFEx v7.2.0 [41] software.

### Feature Extraction

In order to extract radiomic features in LIFEx v7.2.0 software [41], which is compliant with the image biomarker standardization initiative (IBSI) were used [42, 43]. All echo images, including ED and ES images, were resampled (in two-dimensional space with 1 mm intervals) and intensities were quantized to 64 fixed bin number discretized gray levels. Because image intensities range between 0 and 255 values and are constant in all images, this value equals 4 fixed bin size gray levels, which results in 64 ray levels. Finally, 54 features were extracted from each ES and ED image using LIFEx v7.2.0 software. The features included different types of first order (*n* = 21), shape (*n* = 1), and texture ((*n* = 32), GLCM = 7, GLRLM = 11, NGLDM = 3, GLZLM = 11). After extracting the features and classifying the data into two classes (class 1 representing the patients and class 0 representing the control group), the data were divided into two categories: the train (75%, 36 class 0 and 33 class 1) and the test (25%, 11 class 0 and 11 class 1) using stratification.

### Feature Selection

Before FS and the implementation of ML algorithms, some preprocessing on the train dataset was implemented. The radiomic features with zero variance were removed, and correlation between features was investigated using Spearman’s statistical analysis. One of the two features that had an absolute correlation coefficient > 0.90 with each other was removed. Then, feature standardization was done using *Z*-score. In more detail, the training dataset was standardized, and the derived mean and standard deviation (SD) were applied to the test dataset. Analysis of variance (ANOVA), maximum relevance-minimum redundancy (MRMR), and recursive feature elimination (RFE) feature selection methods were used to select the features. Random forest is used as the core of the RFE feature selection approach because it is flexible, resistant to overfitting, computationally efficient, and produces feature importance scores [44].

### Machine Learning

After selecting a certain number of features as input to the models, various types of models such as K-nearest neighbors (KNN), logistic regression (LR), multi-layer perceptron (MLP), random forest (RF), support vector machine (SVM), and eXtreme gradient boosting (XGB) were applied to the data. The architecture of MLP can be found in the supplementary under the heading “The Multi-Layer Perceptron (MLP) architecture”. The data were divided into three categories to feed the ML algorithms: first, only the radiomic features extracted from ED images; second, the radiomic features extracted from ES images; and finally, the combination of the features of these images (ED and ES). Considering 3 separate datasets, 3 FS methods, and 6 different classifiers, a total of 54 models were implemented in this study. GridSearch was used to optimize hyperparameters in the training dataset with 10-fold cross-validation. The best hyperparameters were chosen for the trained model, which was then applied to the test dataset using 1000 bootstraps.

### Model’s Evaluation

Various metrics including the area under the receiver operating characteristic (ROC) curve (AUC), accuracy, specificity, and sensitivity were used to evaluate the performance of the models. In addition, all model’s AUCs were compared using the DeLong test [45]. The DeLong test is designed to compare the ROC curves of two models. It is used to examine whether the difference in AUC between two models is statistically significant, which shows one is more accurate or dependable in a certain situation. The test considers the data’s paired nature and produces a *P* value to estimate the significance of the observed difference [45]. *P* values under 0.05 were regarded as statistically significant. Furthermore, feature maps of four of the most selected features with the highest scores across the three different FS methods were designed for two cases, including a case from the control group (T2* > 20 ms) and a case from the group of patients prone to develop iron overload (T2* ≤ 20 ms). The Pyradiomics version 3.0.1 [42] was used to generate the feature maps, with a kernel radius of 2, the initial value of the feature maps of 0. Moreover, the convolution operation was executed on batches of 10,000 voxels, utilizing the voxelBatch parameter. FS, classification, and statistical test were performed in R version 4.0 [46] using “mlr” [47], “ggplot2” [48], “caret” [49], and “praznik” [50] libraries.

## Results

### Selected Features

**Fig. 2:**
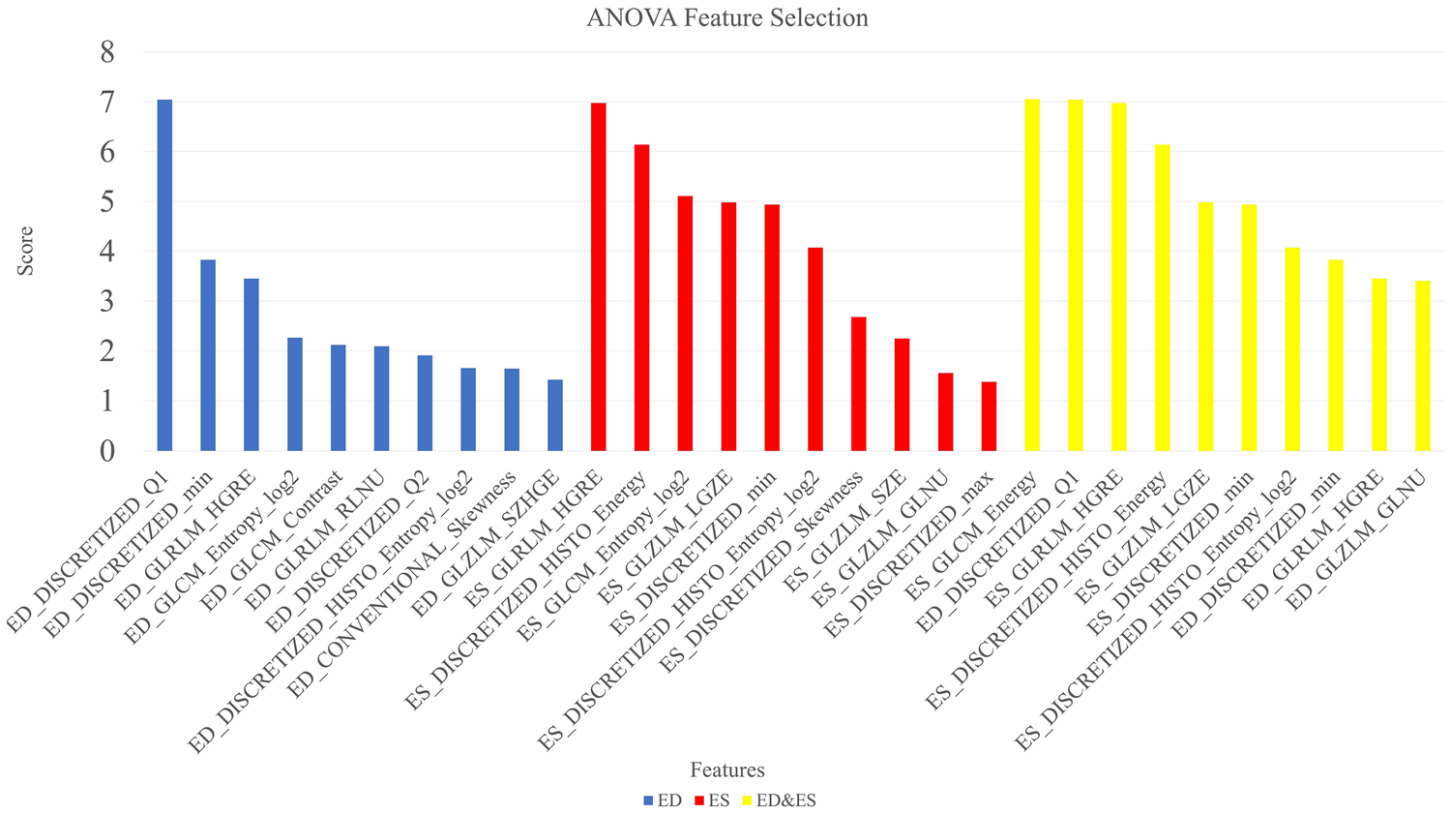
Feature selection using the ANOVA method based on scoring

**Fig. 3:**
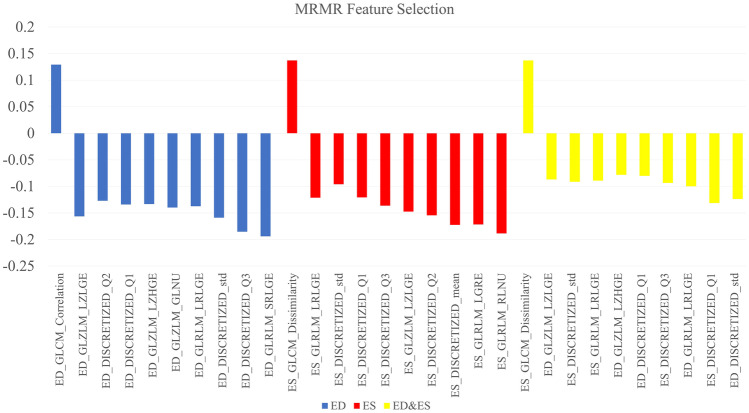
Selected features using the MRMR method and their related scores

**Fig. 4:**
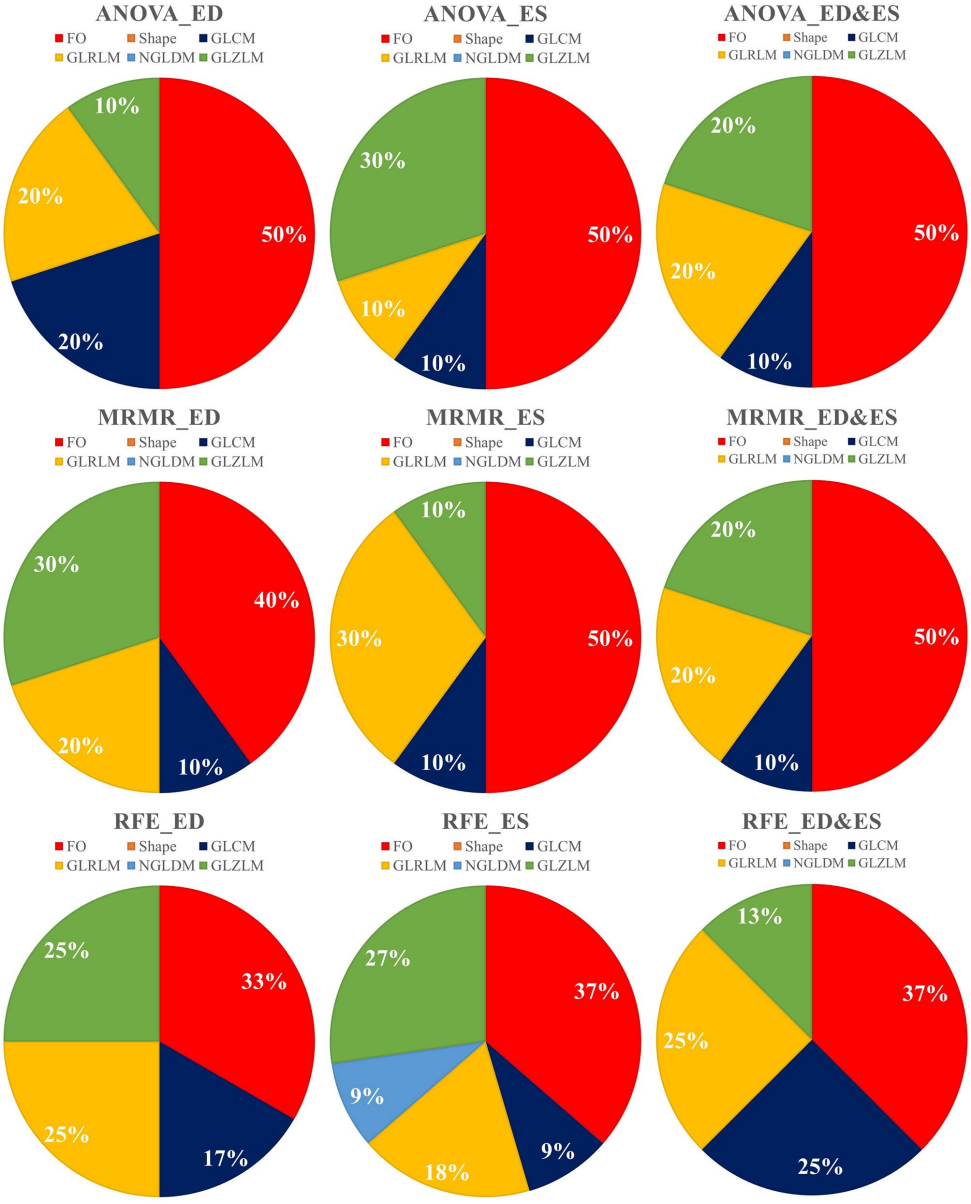
Distribution of radiomic features selected by three feature selection methods including ANOVA, MRMR, and RFE in three different datasets (ED, ES, ED + ES)

Three feature selection methods including ANOVA, MRMR, and RFE were applied on the three datasets (ED, ES, ED + ES), and 10 features were selected in all the datasets for the ANOVA and MRMR methods. Moreover, for RFE, 12, 11, and 8 features were selected in ED, ES, and ED&ES datasets, respectively. Selected features with their scores for ANOVA and MRMR, the linear diagram, and selected features related to the RFE are presented in Figs. 2, 3, 2S, and Table 2, respectively. Also, the distribution of selected features in different modes is plotted as a pie chart in Fig. 4. According to the pie charts, the first-order features had the largest share in different modes, and the shape and NGLDM had the least share.

**Table 2:** The selected features using RFE in different datasets

**ED**	**ES**	**ED&ES**
ED_CONVENTIONAL_Skewness ED_DISCRETIZED_HISTO_Entropy_log2 ED_DISCRETIZED_mean ED_DISCRETIZED_Q1 ED_GLCM_Contrast ED_GLCM_Entropy_log2 ED_GLRLM_HGRE ED_GLRLM_LGRE ED_GLRLM_RLNU ED_GLZLM_LZLGE ED_GLZLM_SZE ED_GLZLM_SZHGE	ES_CONVENTIONAL_max ES_CONVENTIONAL_Q1 ES_DISCRETIZED_HISTO_Energy ES_DISCRETIZED_std ES_GLCM_Entropy_log2 ES_GLRLM_HGRE ES_GLRLM_LRHGE ES_GLZLM_LGZE ES_GLZLM_LZLGE ES_GLZLM_SZE ES_NGLDM_Busyness	ED_GLCM_Contrast ED_GLRLM_HGRE ED_GLZLM_GLNU ES_DISCRETIZED_HISTO_Energy ES_DISCRETIZED_mean ES_DISCRETIZED_std ES_GLCM_Energy ES_GLRLM_HGRE

### Models’ Performance

**Fig. 5:**
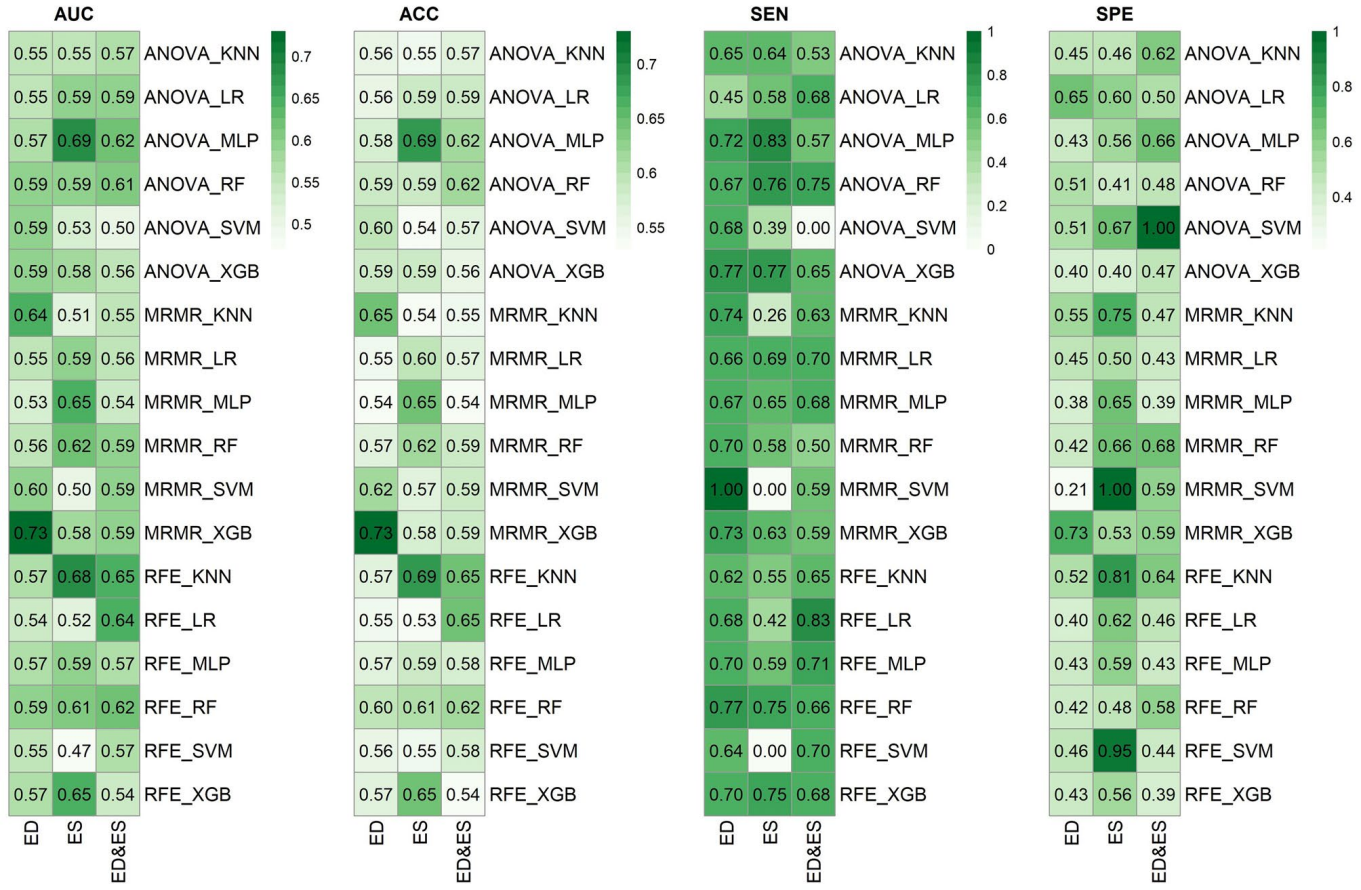
Performance of ML models in different settings including different datasets, feature selection methods, and classifiers. ACC: accuracy, SPE: specificity, SEN: sensitivity, KNN: K-nearest neighbors, LR: logistic regression, MLP: multi-layer perceptron, RF: random forest, SVM: support vector machine, XGB: eXtreme gradient boosting, ANOVA: analysis of variance, MRMR: maximum relevance-minimum redundancy, RFE: recursive feature elimination, ED: end-diastolic, ES: end-systolic

**Fig. 6:**
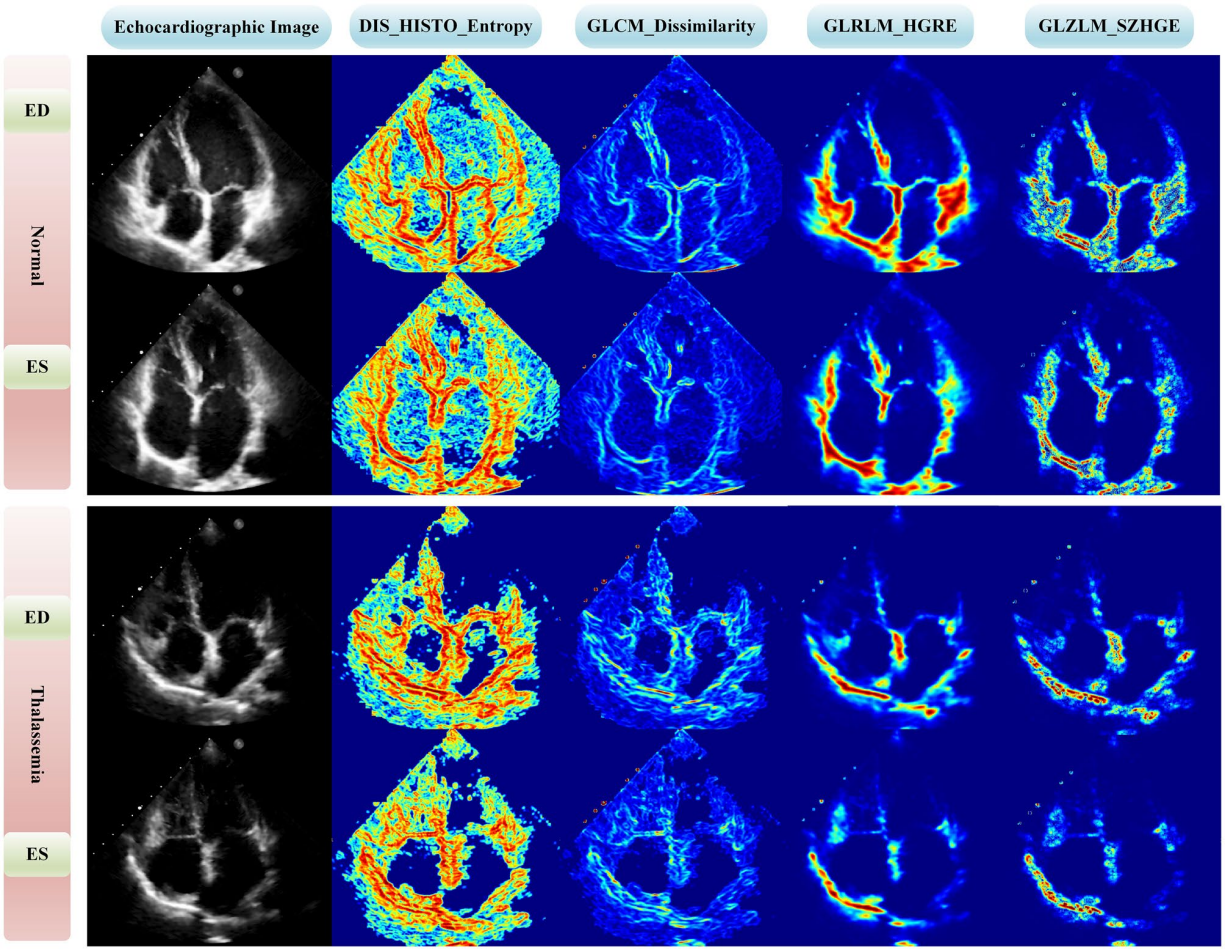
Comparison of normal and thalassemia cases using a feature map approach in four different features to visualize voxel-wise radiomic feature. ED: end-diastole, ES: end-systole

The results of all the models used using six classifiers (KNN, LR, MLP, RF, SVM, and XGB) and three FS (ANOVA, MRMR, and RFE) on three separate datasets (ED, ES, and ED&ES) are shown in Fig. 5. In addition, the performance of the models is evaluated using different criteria of AUC, ACC, SEN, and SPE. The best models in ED, ES, and ED&ES datasets were MRMR-XGB (AUC = 0.73, ACC = 0.73, SPE = 0.73, SEN = 0.73), ANOVA-MLP (AUC = 0.69, ACC = 0.69, SPE = 0.56, SEN = 0.83), and RFE-KNN (AUC = 0.65, ACC = 0.65, SPE = 0.64, SEN = 0.65), respectively. Models with the best performance are listed in Table 3. In addition, the adjusted range for the hyperparameters of the different models used in this study is given in Table 1S in the supplementary section. At last, a feature map comparing a normal and thalassemia case in ED and ES datasets in four features among the best and most selected features according to the FS methods evaluated in this study was generated (Fig. 6).

**Table 3:** The best machine learning models

**Dataset**	**Model**	**AUC ± SD** CI	**ACC ± SD** CI	**SEN ± SD** CI	**SPE ± SD** CI
ED	MRMR_XGB	0.73 ± 0.096 0.72–0.73	0.73 ± 0.094 0.72–0.73	0.73 ± 0.140 0.72–0.73	0.73 ± 0.140 0.72–0.74
ES	ANOVA_MLP	0.69 ± 0.091 0.69–0.70	0.69 ± 0.092 0.69–0.70	0.83 ± 0.110 0.82–0.83	0.56 ± 0.150 0.55–0.57
ES	RFE_KNN	0.68 ± 0.092 0.68–0.69	0.69 ± 0.094 0.68–0.69	0.55 ± 0.150 0.54–0.56	0.81 ± 0.120 0.81–0.82
ED&ES	RFE_KNN	0.65 ± 0.092 0.64–0.65	0.65 ± 0.088 0.64–0.65	0.65 ± 0.140 0.64–0.66	0.64 ± 0.150 0.63–0.65
ES	MRMR_MLP	0.65 ± 0.093 0.64–0.66	0.65 ± 0.090 0.64–0.66	0.65 ± 0.150 0.64–0.66	0.65 ± 0.140 0.64–0.66

*SD* standard deviation, *CI* confidence interval, *ACC* accuracy, *SPE* specificity, *SEN* sensitivity

### DeLong Test

**Fig. 7:**
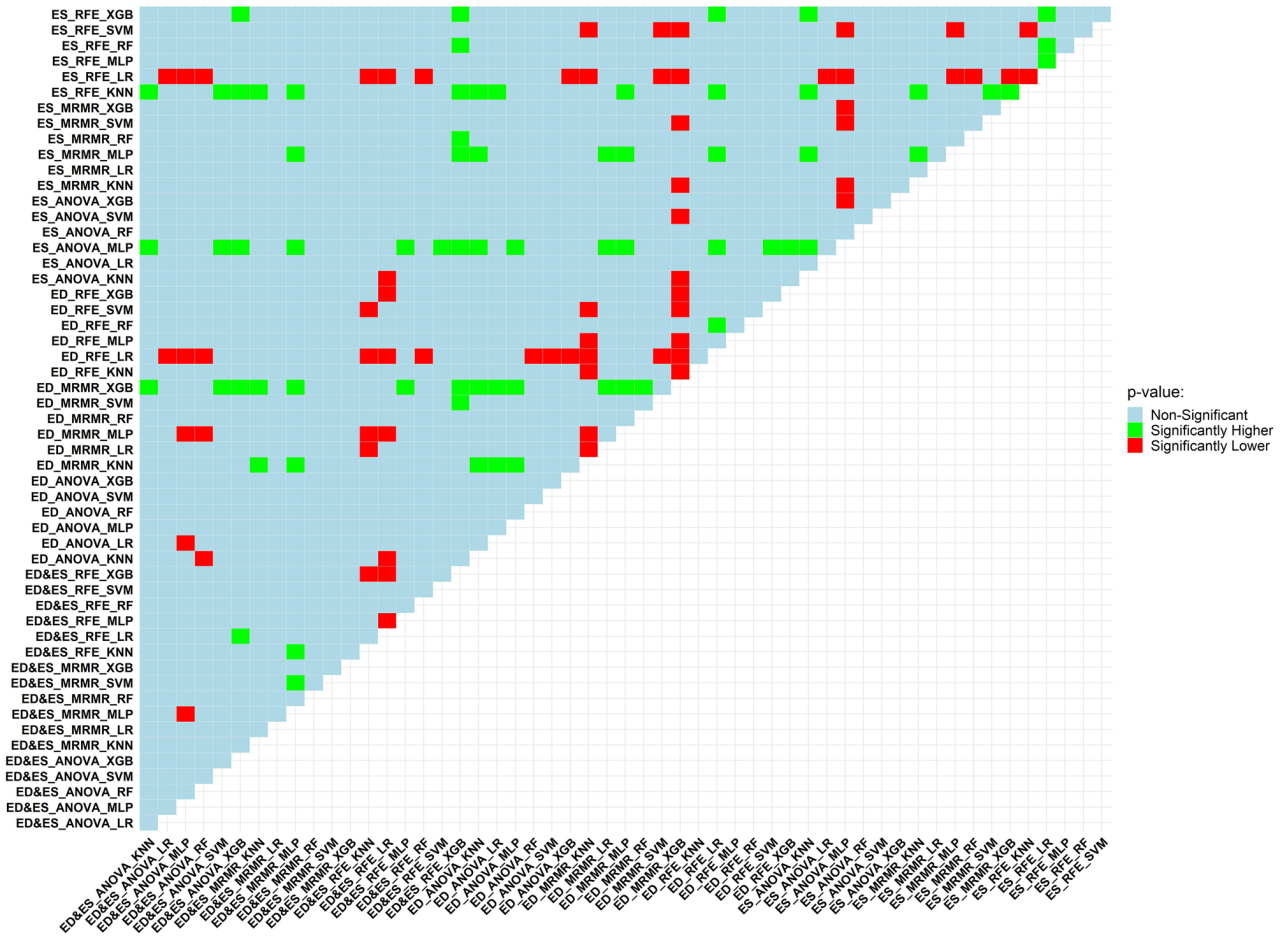
Model performance is compared using the DeLong test, which is run on the models’ AUCs. In this figure, the models on column and row were evaluated against each other. Green, if the row model considerably outperformed the column model in terms of *P* value; red, if the row model’s *P* value was much lower than the column model’s. If the comparison between the row model and column model yielded a non-significant *P* value, light blue would be the color

Figure 7 presents the DeLong test findings. A total of 54 different models were implemented in this study, and the AUC of all these models was compared with each other using the DeLong test. The outcomes were categorized as non-significant and statistically significant (substantially lower or higher). ED-MRMR-XGB, ES-ANOVA-MLP, and ES-RFE-KNN models were among the best models of this study. They had 24, 21, and 16 statistically higher *q* values compared to other models, respectively.

## Discussion

Despite recent developments, heart failure resulting from iron deposition in patients with thalassemia major is still the most serious and the leading cause of death [18]. Furthermore, patients with thalassemia major may not have any symptoms, which can delay the early diagnosis of myocardial dysfunction and put successful reverse disease conditions at risk [10]. Iron overload identification methods such as serum ferritin and liver and heart biopsy have limitations and cannot be used as a reliable and accurate method to evaluate myocardial iron concentration [15]. T2*CMRI is an outstanding and non-invasive diagnostic technique in identifying cardiac iron content [10], although obstacles such as being expensive and time-consuming, not being generally available in all medical centers, and the presence of contraindications for MRI prevent its widespread use [18]. Echocardiography can also evaluate heart failure resulting from iron deposition. Availability, outpatient, and portable are the advantages of echo; nevertheless, the interpretation of this method highly depends on the user’s knowledge and experience [24]. In this study, the CMRI findings are utilized to categorize participants into two groups: normal and prone to thalassemia. It should be emphasized that while echo results were comparable (LEFV > 55%) in all patients, those with T2* ≤ 20 ms were more likely to experience future cardiac issues. In order to categorize patients and determine who is most likely to experience cardiac difficulties owing to iron overload in the future, radiomic features of echo images were used. As was already noted, early identification of these individuals can have a crucial role in the treatment process and reducing the mortality rate. To the best of our knowledge, this study is the first attempt to identify cardiac problems caused by iron overload using radiomic features extracted from echo images and ML based on T2* values obtained from MRI. In previous studies [6, 8–10, 17, 18, 51], statistical tests and software determined the correlation between T2* and echo parameters.

According to Fig. 4, first-order, GLRLM, and GLZLM were the most frequent features in three FS methods. In detail, in the ANOVA method, first-order (50%) and GLZLM (20%) features; in the MRMR method, the first-order (47%) and GLRLM (23%) features; and in the RFE method, the first-order (36%) and GLRLM (23%) features were the most frequent. Shape features were not selected in any FS methods because these features have no relationship with the amount of iron deposition and the amount of T2* in the septal area. Among the features selected by the ANOVA method, the highest scores in ED, ES, and ED&ES datasets belonged to the first quartile of discretized (DISCRETIZED_Q1), GLRLM_High Gray-Level Run Emphasis (HGRE), and GLCM_Energy features, respectively (Fig. 2). Joint Energy as one of the GLCM features evaluates the homogeneity patterns in the myocardium [31] and HGRE as GLRLM feature determines the distribution of the higher gray level values [43]. An effective substitute for the coefficient of variance is the quartile coefficient of dispersion. The first quartile (Q1) also evaluates the distribution of gray level values [43]. Then, among the features selected by the MRMR method, the highest scores in ED, ES, and ED&ES datasets belonged to the GLCM_Correlation, GLCM_Dissimilarity, and GLCM_Dissimilarity features, respectively (Fig. 3). Correlation as GLCM feature measures the linear dependency of gray levels and dissimilarity shows the local intensity variation [42]. Although, as in CMRI images, iron deposits cause the myocardium containing iron overload to have a lower signal and intensity compared to normal tissue [11], in echocardiography images, iron accumulation leads to the heterogeneous distribution of the intensity of gray levels. These radiomic features will help identify patients without any heart failure.

Barzin et al. [18] stated that all diastolic functional indicators, except for early (E) and late (A) transmitral peak flow velocity ratio (E⁄A), exhibit a notable relationship with T2*. In our research, the radiomic features showed that diastolic indices are related to the T2* parameter. Meanwhile, in the study of Aypar et al. [10], diastolic dysfunction was seen locally in the septal wall in patients with thalassemia major. In our study, the radiomics from diastolic were obtained from the segmentation area (septum) and had the highest score and importance in the ANOVA method.

Model explainability seeks to identify a distinctive set of biomarkers, known as a signature, to potentially predict a clinical outcome, such as a diagnosis, prognosis, or response to treatment. In the realm of radiomics, intriguing research has been carried out recently. However, there is a lack of emphasis on developing explainable models. The essence of explainable models lies in their ability to gain approval and trust from physicians in clinical setting. When a model is developed, it becomes crucial to demonstrate to physicians that it is not just a black-box computerized system. By providing explanations for the model’s outcomes, it can foster confidence and encourage the utilization of these models in practice [39].

The important issue is that many of the developed models are not easily interpretable. Physicians and clinicians cannot easily understand and subsequently trust them because of their black-box nature [39]. In Fig. 6, the four features that had the highest scores and the most selections among different FS methods were visualized by voxel-wise feature extraction for different classes. Entropy, from first-order features, provides randomness of the intensity distribution in the region of interest (ROI). A lower entropy value denotes a more uniform distribution, while a greater value reflects a more heterogeneous intensity [43, 52]. Dissimilarity, a GLCM-derived feature, measures the difference between adjacent pixel intensities, revealing changes in intensity values and indicating texture edges or sharp transitions. Higher dissimilarity values indicate greater contrast and variation, while lower values suggest more uniformity [43, 52]. HGRE, a GLRLM-derived feature, shows the image frequency and length of runs of consecutive pixel values. It measures the importance or weighting of the image’s longer runs with higher gray-level values and emphasizes the dominance or prevalence of runs with high values for the gray level [43, 52]. Small Zone High Gray-Level Emphasis (SZHGE), a GLZLM-derived feature, evaluates the significance or prioritization given to smaller zones containing higher gray-level values within the image. It offers insights into these small zones’ frequency and dominance, characterized by elevated gray-level values [43, 52]. The mean value of entropy and GLCM dissimilarity was higher, and the mean value of GLRLM_HGRE and GLZLM_SZHGE was lower in the control group both in ES and ED datasets. Our hypothesis regarding the former two features is that the formation of iron overload may have reduced the amount of dissimilarity and randomness of the intensity, which caused these values to be lower in patients compared to the control group. In terms of the latter two features, it can be hypothetically related to the points where iron overload is developing.

In the ED dataset, the MRMR-XGB model achieved the best result. In the ES dataset, the top models were MLP using ANOVA and KNN using RFE. In the ED&ES dataset, RFE-KNN had the best result. The results of the models in the ED dataset are superior to those of each set of features. The explanation is that the motion of the heart is the lowest in the mid-to-end-diastolic phase; this probably causes the distortion of the features to occur less and get a better result.

According to our findings, using radiomics extracted from echo images, it is possible to classify individuals which are labeled according to CMRI T2*. Meanwhile, the subjects examined in this study had normal results in terms of LVEF, and no dysfunction was evident. In other words, based on image analysis, echo radiomic features are related to the T2* value. While in conventional echocardiography studies, Moussavi et al. [21] found no remarkable association between T2*MRI and echocardiographic results. Vogel et al. [9] stated that the sensitivity of tissue doppler echocardiography in detecting abnormal iron load is 88%, and its specificity is 65%. In contrast, 73% of sensitivity and 73% of specificity in the MRMR-XGB model and 83% sensitivity and 56% specificity in the ANOVA-MLP model were achieved in our study. Aypar et al. [10] also concluded that when the mid-septal Sm ≤ 5.7 cm⁄s, the tissue Doppler echocardiography sensitivity is 63%, and the specificity is 83%, and when the mid-septal Em ≤ 12.1 cm⁄s, the sensitivity is 75%, and the specificity is 75%. Djer et al. [17] claimed no significant relationship exists between T2* and left ventricular systolic indices. While in our study ANOVA-MLP among the models applied on the ES dataset (AUC: 0.69, SPE: 0.56, SEN: 0.83, and ACC: 0.69) had the best performance in diagnosis of cardiac problems caused by iron overload. ANOVA-MLP is considered among the top three models. Since systolic dysfunction occurs late in the disease process, this finding can be significant.

Our study emphasizes the high ability of radiomics in the early detection of cardiomyopathy resulting from iron deposition in conditions where LVEF is preserved. Therefore, the presented findings could potentially help physicians make decisions regarding heart failure caused by iron deposition using echo images. In such a way, physicians can successfully reverse the condition of cardiomyopathy and prevent the progression of the disease with early diagnosis. Furthermore, since echocardiography has a lower cost than a method like MRI and is available in most centers, this method is cost-effective in evaluating heart failure in patients with thalassemia. In addition, echocardiography is non-invasive as well as portable.

This study had some limitations. First, we have a small sample size as we select patients with echo and CMRI studies in short time intervals with a max 6-month duration; a larger sample in future studies would be of more value. In this study, data were collected from one center. To ensure the models’ generalizability, collecting data from different centers and evaluating model performance across different centers is necessary. As RFE only benefited from the RF model, it is possible that the features selected may not be the most optimal choice for other classifiers.

## Conclusion

The ED-MRMR-XGB has presented promising and acceptable results among ML algorithms. According to the results of the echo images, the individuals in the study had the same conditions (LVEF > 55%), but they were different based on the CMRI results, which were labeled accordingly, and then using the radiomic features extracted from the echo images and ML approaches were classified. Although the results of LVEF from echo were similar, by using the radiomic features extracted from these images, our models obtained promising results, which indicate that with non-invasive, inexpensive, portable, and highly accessible echocardiography, it is possible to identify who is prone to suffer from heart problems caused by iron overload in the near future and this can lead to a proper treatment to prevent cardiac problems and death of these patients. Therefore, early diagnosis of heart failure, even before the appearance of symptoms, has been made possible by radiomics of echo images and ML.

### Supplementary Information

Below is the link to the electronic supplementary material.

Supplementary file1 (DOCX 2040 KB)

## Data Availability

Not applicable.

## Abbreviation

CMRI: Cardiac magnetic resonance imaging
ML: Machine learning
LVEF: Left ventricular ejection fraction
ES: End-systolic
ED: End-diastolic
FS: Feature selection
AUC: Area under the ROC curve
SEN: Sensitivity
SPE: Specificity
ACC: Accuracy
SD: Standard deviation
SSFP: Steady-state free precession
IBSI: Image biomarker standardization initiative
ANOVA: Analysis of variance
MRMR: Maximum relevance-minimum redundancy
RFE: Recursive feature elimination
KNN: K-nearest neighbors
LR: Logistic regression
MLP: Multi-layer perceptron
RF: Random forest
SVM: Support vector machine
XGB: eXtreme gradient boosting

## Code Availability

LIFEx v7.2.0 and Python libraries used in this study.
