# Supplementary material for: Left Ventricular Myocardial Dysfunction Evaluation in Thalassemia Patients Using Echocardiographic Radiomic Features and Machine Learning Algorithms
Source: J Digit Imaging. 2023 Sep 21;36(6):2494–506. doi: 10.1007/s10278-023-00891-0 (PMC10584796; doi:10.1007/s10278-023-00891-0)
Supplement: Supplementary file 1 — Supplementary file1 (DOCX 2040 KB) [file 10278_2023_891_MOESM1_ESM.docx]

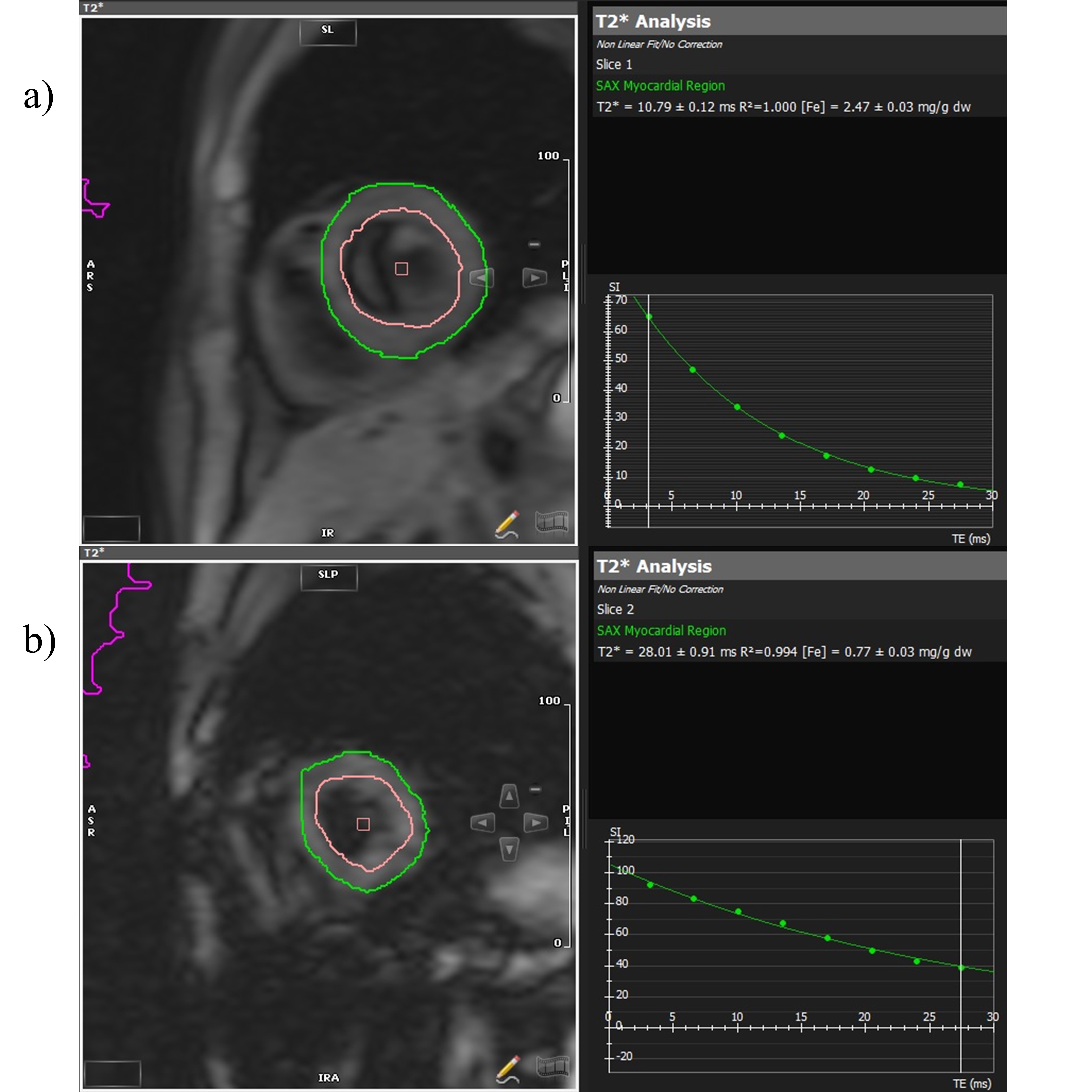


**Fig. 1S** CMRI images of two different cases are displayed. a) A case where T2* ≤ 20 ms can be seen, which indicates iron overload in heart. b) A heart image of a person whose T2* > 20 ms can be seen.


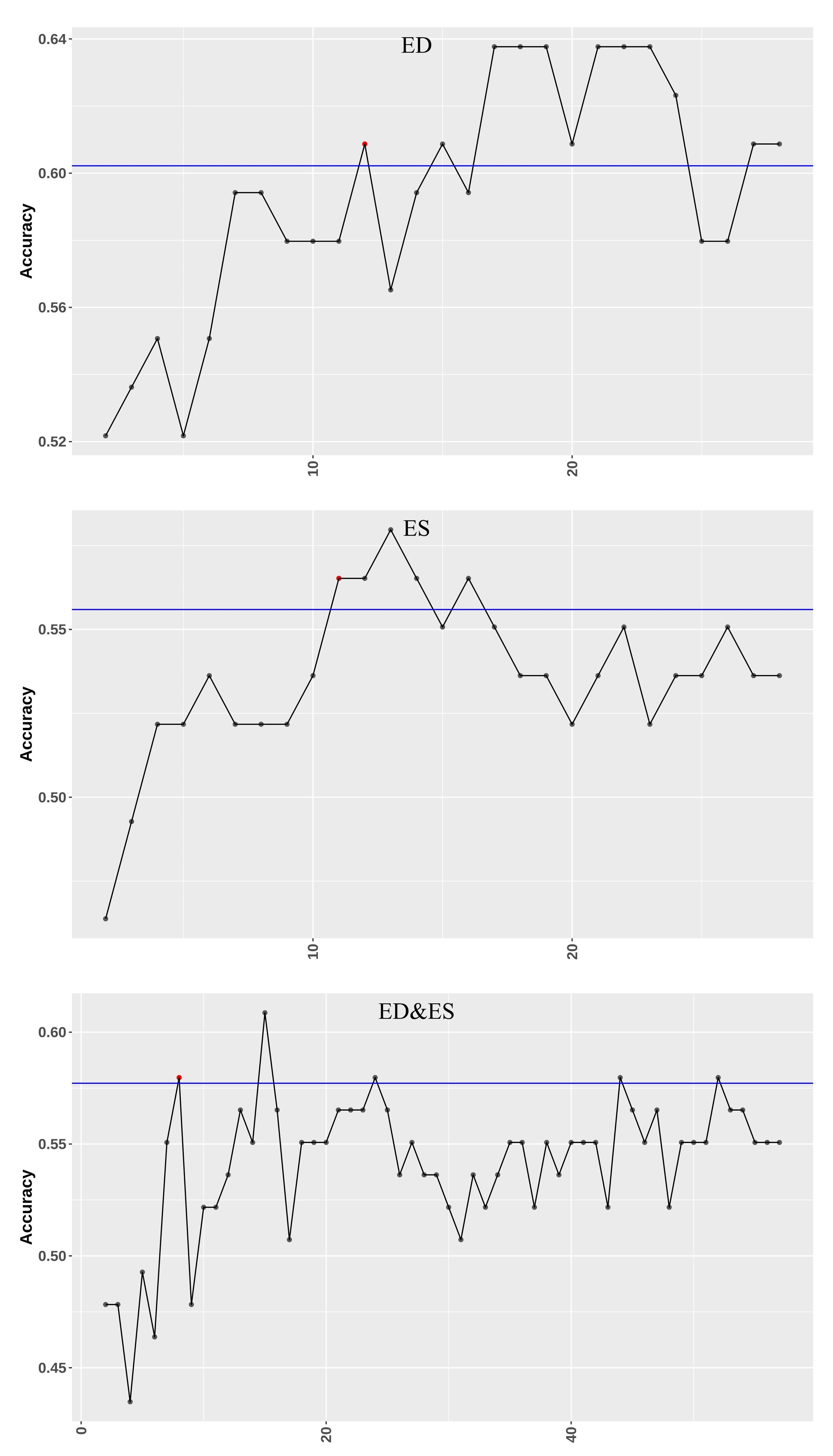


**Fig. 2S** The diagram related to the feature selection process by RFE. The number of selected features is shown using a red index.

**Table 1S**. hyperparameters’ range in different classifiers for optimization.

| Classifier | Hyper-parameter | Range |
| --- | --- | --- |
| XGB | eta | 0.025, 0.05, 0.1, 0.3 |
|  | max_depth | 2:10, step=1 |
|  | nrounds | 50:1000, step=50 |
|  | colsample_bytree | 0.4, 0.6, 0.8, 1.0 |
|  | subsample | 0.5, 0.75, 1.0 |
|  | gamma | 0, 0.05, 0.1, 0.5, 0.7, 0.9, 1.0 |
|  | min_child_weight | 1, 2, 3 |
| SVM | cost | 0.1:10, step=0.1 |
|  | gamma | 0.1:10, step=0.1 |
| KNN | k | 1:12, step=1 |
| RF | ntree | 50:1000, step=50 |
|  | mtry | 1:10, step=1 |
|  | nodesize | 1:20, step=1 |
| LR | - | - |
| MLP | size | 1:10, step=1 |

**The Multi-Layer Perceptron (MLP) architecture**

The MLP architecture was composed of three layers: an input layer, a single hidden layer, and an output layer. During the hyperparameter optimization process, the number of units in the hidden layer was treated as a hyperparameter and optimized accordingly. The training process was limited to a maximum of 100 iterations, as specified by the “maxit” parameter. The initial weights for the network were randomized using the “Randomize_Weights” initialization function, with parameters set between -0.3 and 0.3. The learning function employed was the “Std_Backpropagation” algorithm, utilizing a learning rate of 0.2 and a momentum of 0. The weights were updated using the “Topological_Order” method, which updates the weights based on the order in which the layers are connected. The activation function “Act_Logistic” was used for both the hidden and output units. During training, the training patterns were randomly shuffled before each epoch, as indicated by the “shufflePatterns” parameter set to TRUE. Additionally, the output units used the logistic activation function, as the “linOut” parameter was set to FALSE. No separate validation set was used during training, as the “inputsTest” and “targetsTest” parameters were set to NULL. Furthermore, the “pruneFunc” and “pruneFuncParams” parameters were not utilized in the training process.
